# The Economic Impact of Clinical Research in an Italian Public Hospital: The Malignant Pleural Mesothelioma Case Study

**DOI:** 10.15171/ijhpm.2018.13

**Published:** 2018-02-18

**Authors:** Roberto Ippoliti, Greta Falavigna, Federica Grosso, Antonio Maconi, Lorenza Randi, Gianmauro Numico

**Affiliations:** ^1^Scientific Promotion, General Hospital of Alessandria, Alessandria, Italy.; ^2^Department of Management, University of Turin, Turin, Italy.; ^3^Research Institute on Sustainable Economic Growth, National Research Council of Italy, Moncalieri, Italy.; ^4^Oncology Unit, General Hospital of Alessandria, Alessandria, Italy.

**Keywords:** Rare Tumors, Clinical Research, Budget Constraint, Health Planning, Malignant Pleural Mesothelioma

## Abstract

**Background:** The current economic constraints cause hospital management to use the available public resources as rationally as possible. At the same time, there is the necessity to improve current scientific knowledge. This is even more relevant in the case of patients with malignant pleural mesothelioma (MPM), given the severity of the disease, its dismal prognosis, and the cost of chemotherapy drugs. This work aims to evaluate the standard cost of patients with MPM, supporting physicians in their decision-making process in relation to budget constraints, as well as policy-makers with respect research policy.

**Methods:** The authors conducted a retrospective cost analysis on all the patients with MPM who were first admitted to a reference hospital specialized in MPM care between 2014 and 2015, collecting data on their diagnostic pathways and active treatments, as well as on the related official fees for each procedure. Then, using a multiple regression model, we estimated the overall expected cost of a patient with MPM treated in our hospital, to be born by the Regional Healthcare System based on the chosen clinical pathway.

**Results:** According to results, the economic impact of caring for a patient with MPM is mostly related to the selected active treatments, with drug and hospitalization costs as main drivers. Our analysis suggests that the expected reimbursed fee to care for a patient with MPM is equal to € 18 214.99, with chemotherapy and monitoring costs equal to € 12 861.43 and hospitalization cost equal to € 5353.55. This cost decreases to € 320.18 in the case of enrollment in an experimental trial of first-line treatment. In the other cases (second-line or third-line trials), the expected cost borne by the healthcare system for treating patients grows exponentially (€ 40,124.18 and € 59 839.94, respectively).

**Conclusion:** Experimental trials might be a solution to decrease the economic burden for the public healthcare system only in the case of first-line treatments, where the cost of chemotherapy is relevant. Nevertheless, policy-makers have to accept the sharing of this economic burden between society and the pharmaceutical industry to broaden the current scientific knowledge.

## Background

Malignant pleural mesothelioma (MPM) is an aggressive tumor predominantly arising in the pleural cavity and related to asbestos exposure. Despite the extensive use of asbestos since the end of the Second World War, which has produced a significant increase in incidence and mortality among the population of industrialized countries, MPM is considered a rare tumor, with an estimated incidence of 3.5/100.000/y in males and 1.3/100.000/y in females. However, in contaminated areas, the incidence rises to 10 to 20 times above the expected rate. MPM prognosis is very poor and average survival is between 9 and 12 months after diagnosis.^1^

Italy was an important asbestos producer and user until its prohibition in 1992, with a peak of about 3 748 550 tons of row asbestos consumption between 1976 and 1980. In particular, asbestos was used in several industrial activities, such as the chemical industry, steel industry, metalworking, construction of railroad cars and ships, and cement industry.^2^ According to Italian epidemiological studies, the incidence of MPM is due to occupational and non-occupational exposure (eg, environmental and domestic exposure), with an overall higher mortality risk for the population living near asbestos-cement plants, like in the North West of Italy. In particular, one of the main active asbestos-cement factories (ie, Eternit) was located in Casale Monferrato, branding this area as one of the most asbestos polluted. Indeed, this area displays the highest incidence of MPM cases.^3^

MPM and its management are considered a serious public health problem, also representing a major challenge for the finances of the regional public healthcare system. Despite being unsuccessful, the existing chemotherapy treatments are extremely expensive. The current austerity approach only makes it more difficult for hospital managers to keep within their budget constraints while, at the same time, offering an appropriate supply of cancer treatments to patients with MPM. On the one hand, society needs to advance the current scientific knowledge, identifying innovative successful treatments. On the other hand, budget constraints lead hospital managers to search for alternatives to existing, highly priced chemotherapy, which is even more relevant if we consider the current worldwide period of austerity and the widespread spending review policy in the healthcare sector.^4-6^ Is it admissible to believe that experimental activities might represent a solution to these open issues?

According to the current literature, participating in clinical trials would yield clear benefits such as, for example, contributing to the advancement of medical research and/or ensuring access to experimental treatments,^7,8^ although patients would have to share a portion of the risks related to these innovative treatments.^9^ Obviously, this is extremely significant in the case of MPM, ie, one of those pathologies for which there are no effective clinical alternatives.^10^ Another key reason for participating in clinical trials is that they eliminate drug expenditure, which might have a significant economic impact on the whole healthcare system,^11^ its hospitals,^12,13^ pharmacies,^14,15^ as well as oncology units.^16^ Indeed, drugs might be supplied for free by the sponsor of the clinical trial, thus bringing about a substantial saving, which is even more significant considering the increasing price of chemotherapy.^17-19^ However, there is also evidence against this hypothesis, since clinical trials might result in a heavier clinical and administrative burden, with a negative economic impact on the budget.^20-22^

Indeed, when a patient is enrolled in a clinical trial, specific medical procedures are necessary (ie, additional checkups, blood tests and radiological exams), as well as other non-treatment trial activities.^16,20^ This means that the principal investigator and his/her collaborators need not only time and effort to correctly manage all the duties required by the experimental protocol but also additional economic resources. This is why pharmaceutical companies provide grants as a form of economic compensation for these operating costs, even though they might not be sufficient. Researchers identify higher expenditure as the main reason for the non-activation of trials, reflecting the rising costs and complexity of cancer clinical trials.^23^ Moreover, the unexpected toxicity of experimental drugs might increase the probability of hospitalization, with a significant growth in public expenditure.

Nevertheless, little research has been carried out on the costs of treating patients within clinical trials, taking the whole clinical pathway into account. On the one hand, the current literature compares experimental treatments (with free drugs) to alternative standard treatments (involving a cost), but operating costs are not taken into account. On the other hand, researchers focus exclusively on operating costs due to the higher number of medical activities required by experimental protocols. In both cases, the whole clinical pathway is not taken into account. In other words, the current literature does not indicate whether drug cost avoidance (plus a grant) might offset the expected higher operating costs and the unexpected toxicity, considering the whole clinical path of these patients (ie, from their first access to their death). A micro analysis is necessary to properly compare alternative clinical pathways and, in this way, shed new light on this key issue.

This work aims to compare clinical pathways, focusing on the economic impact of experimental and standard treatments on the healthcare system. Standard treatments are understood as all those treatments suggested by national and international guidelines based on collected clinical evidence, ie, the current best answer to MPM, while experimental treatments are all those innovative treatments which need clinical evidence to be accepted by the scientific community and authorized by the National Drug Agency. In particular, although experimental treatments can provide innovative ways to cure patients, the authors investigate the hypothesis that the economic costs needed to treat research subjects might actually be borne also by the public healthcare system. In other words, we test whether clinical trials might represent a way to save public resources or whether there are economic costs which the hospital management has to bear to improve scientific medical knowledge. The results are not easily predictable since clinical trials might represent a saving due to drug cost avoidance but, at the same time, they might entail higher operating costs.

The structure of this paper is as follows. Section two presents our data and the adopted methodology, while the results of the empirical analysis are set out in section three. Finally, the last section puts forward some conclusions.

## Methods

The authors conducted a retrospective cost analysis on all the patients with MPM first admitted to a reference hospital specialized in MPM care between 2014 and 2015, focusing on those who died before June 2016. Exclusion criteria were only non-epithelioid istotype. We collected data about the patients’ diagnostic pathways and all the treatments provided by the hospital, applying the official regional fees for each procedure to estimate their costs (D.D. June 16, 2015, no. 371). Indeed, this hospital is a public institution and its activity is reimbursed by the regional public healthcare system, according to current regulations and applying the cited price list. Then, using a multiple regression model, we estimated the overall expected cost of treating a patient with MPM in the hospital, which would be borne by the regional healthcare system. Finally, adopting the estimated coefficients, we implemented a simulation to compare clinical pathways with experimental and/or standard treatments.

According to the current national regulations in Italy, patients are free to choose their health provider, without paying a fee (eg, in case of hospitalization) or, in some cases, paying a given fixed contribution (eg, in case of diagnostic exams). Obviously, the patients’ choice is driven by the providers’ efficiency in offering therapeutic treatments, their quality and/or the proposed level of innovation.^24^ Afterwards, the Local Health Authority responsible, on a territorial basis, for the health of those patients will reimburse the health provider for all the treatments recognized as essential by the Italian Ministry of Health (ie, *Livelli Essenziali di Assistenza*). In other words, each of the regional healthcare systems has to guarantee its citizens some specific treatments, which might be bought on the market or provided directly by the Local Health Authority, with a reimbursement system that should lead to increased levels of efficiency.^25-27^

In our specific case study, an independent public hospital supplies treatments to patients with MPM free of charge (ie, these treatments are recognized as essential by the Italian Ministry of Health) and the Local Health Authority then reimburses these activities according to pre-established standard fees (ie, treatments are bought on the market at a fixed price). These fees have been adopted by the authors to estimate the expected costs of treating patients according to different clinical pathways, considering all activities. Obviously, the proposed fees might be higher or lower than the real costs borne by the analyzed independent public hospital but, based on the current reimbursement system and the suggested idea of efficiency, we can expect general convergence among all health providers toward a certain value. In other words, this work assumes a competitive market equilibrium in the long run with no profit, that is to say, real costs equal to standard fees.^28,29^ Therefore, considering our case study, patients with MPM do not bear the cost of the treatments, that is to say, there is no income effect behind their decisions concerning treatment. Their choices are clearly driven by the opportunity to receive innovative (experimental) treatments with greater expected effectiveness.^24^

Table 1 summarizes antitumoral treatments offered to patients with MPM in the selected hospital, highlighting the different clinical pathways (ie, standard treatments and/or alternative experimental strategies).

**Table 1:** Potential Clinical Pathways for Patients With MPM

**Line of Chemotherapy**	**Standard Treatment**	**Experimental Treatment**^a^
First line	Cisplatin and pemetrexed *or* carboplatin and pemetrexed	NCT01907100 (First-line study)
Maintenance line	-	NCT01907100 or NCT01358084 (Maintenance study)
Second line	Gemcitabine *or* vinorelbine *or* carboplatin and gemcitabine	NCT01843374 (Second-line study)
Third line	Gemcitabine *or* vinorelbine	NCT02194231 (Third-line study)

Abbreviation: MPM, malignant pleural mesothelioma. ^a^ See clinicaltrials.gov.

Obviously, the decision to opt for an experimental treatment has to be agreed with the patient but the preliminary step (ie, proposing alternatives) is a prerogative of the physician. Depending on the patient’s characteristics and his/her expected side effects tolerability, the physician will suggest the best option for each line. This clinical ex-ante evaluation of side effects tolerability is mainly based on age and concomitant diseases (eg, diabetes, neurological conditions, cardiovascular conditions), as well as renal, liver and bone marrow function. For example, cisplatin and pemetrexed might be proposed for first-line treatment (standard treatment), followed by an experimental maintenance study (study NCT01358084), with another standard treatment for the second line (gemcitabine) and third line (vinorelbine). The choice between cisplatin and carboplatin – in combination with pemetrexed – will depend on the physician’s evaluation of expected side effects tolerability. In this example, the choice of cisplatin may be due to better renal function, as suggested by the current literature.^30,31^ Afterwards, the enrollment in the experimental maintenance study (study NCT01358084) will depend on the inclusion criteria set by the sponsor regarding tolerance to the expected toxicity of the innovative drug (ie, research subjects’ side effects tolerability).

Clearly, it is not possible to establish a priori which the entire clinical path might be, since the patient’s response to the therapies ought to be observed step by step. In order to compare observations and estimate whether a significant saving of public resources might really exist, we need to control for patient conditions (eg, cancer stage and/or treatment-related side effects) potentially affecting the main cost components (ie, hospitalization and medical/instrumental examinations). In other words, an in-depth analysis must be carried out adopting more sophisticated statistical tools to estimate whether drug cost avoidance might compensate for the greater burden of activities related to the clinical trials, controlling for some key conditions.

We analyzed the selected sample of patients adopting an ordinary least squares (OLS) regression model to evaluate the main cost components to be borne by the healthcare system for patients affected by MPM, applying the robust option. Additional model specifications are also proposed, such as a sensitivity analysis, dropping variables, and controlling whether the signs of the remaining variables change. The proposed approach is coherent with the current literature,^22^ estimating a statistically significant weight for every cost driver. Then, based on the collected results (ie, the estimated weights), *ceteris paribus*, we compared different clinical pathways, focusing on the expected final cost.

In detail, for every patient, the following control variables are proposed:

- *time (age)*, which is the age of each of the patients at the time of their first medical access (expressed in years);
- *time (survival)*, which is the time between first medical access and death (expressed in months);
- *non-naïve patient*, which is a dummy variable equal to 1 if the patient had already been treated in other medical centers, 0 otherwise;
- *exit patient*, which is a dummy variable equal to 1 if the patient left our hospital to be treated in other medical centers, 0 otherwise;
- *cancer stage*, which is a categorical variable (values from 1 to 4) referring to the extent of MPM;
- *side effects tolerability*, which is a dummy variable equal to 1 if tolerability to the potential side effects of chemotherapy can be expected on the basis of clinical ex-ante evaluations (eg, considering age and concomitant diseases, as well as renal, liver and bone marrow function), 0 otherwise;

while, focusing on the explanatory variables, we adopted:

- *hospitalization*s, ie, the number of hospitalizations in our oncology unit;
- *first line (cycles)*, which is the number of cycles in the first-line treatment, normalized between 0 and 1 (with 6 cycles as the expected maximum number of cycles in this line);
- *maintenance (cycles)*, which is the number of cycles after the first-line treatment in order to maintain the achieved response status (note that this treatment line is available exclusively in experimental strategies);
- *second line (cycles)*, which is the number of cycles in the second-line treatment, after the first-line treatment or after the experimental maintenance treatment (if the patient was involved in that trial);
- *third line (cycles)*, which is the number of cycles in the third-line treatment.

Note that, for the first-line study, we decided to consider the expected number of cycles based on the current literature. Hence, we estimated the number of implemented cycles in relation to the expected number (ie, 6 cycles), so as to consider the percentage of adherence to the current guidelines for first-line studies. Conversely, for what concerns the subsequent lines, there is no standard number of cycles.

After that, considering both the standard and the experimental treatment, in order to add further information to complement the previous variables, we introduced the following additional variables into the model:

- *standard treatment (quota)*, which is the percentage of standard cycles over the total number of cycles supplied to the patient (between 0 and 1);
- *first-line and maintenance study*, which is a dummy variable equal to 1 if the patient was involved as a research subject in the experimental first-line treatment (ie, study NCT01907100), 0 otherwise;
- *maintenance study*, which is a dummy variable equal to 1 if the patient was involved as a research subject in the experimental maintenance treatment (ie, study NCT01358084), 0 otherwise;
- *second-line study*, which is a dummy variable equal to 1 if the patient was involved as a research subject in the experimental second-line treatment (ie, study NCT01843374), 0 otherwise;
- *third-line study,* which is a dummy variable equal to 1 if the patient was involved as a research subject in the experimental third-line treatment (ie, study NCT02194231), 0 otherwise.

In other words, these variables indicate whether the number of cycles, referred to by the previous variables, were supplied within a standard clinical pathway or an experimental pathway.

Obviously, the total amount of Euros which would be reimbursed by the healthcare system to the hospital in charge of looking after patients with MPM (*total cost*) is the dependent variable of our multiple regression model.

Subsequently, using the estimated coefficients of the multiple regression model, we calculated the expected cost of treating the same patient but adopting different clinical pathways, ie, comparing experimental and current treatments. In detail, we considered a patient who is 50 years old, with a stage III disease, not pre-treated and potentially eligible for Cisplatin-based chemotherapy. A further condition is for the entire clinical pathway to be completed in the same medical center. The expected survival time from first medical access is 24 months. Taking the chemotherapy treatments into account, we hypothesized 6 first-line cycles, 4 second-line cycles and, finally, 4 more cycles in the third line. Moreover, when we simulated the involvement of patients in the experimental first-line and maintenance strategies (ie, NCT01907100) as well as in the maintenance line (ie, NCT01358084), we considered an additional 4 maintenance cycles.

The proposed comparative simulation considers 5 different clinical pathways. Case I assumes all standard treatments while the other cases are characterized by a mixed pathway with different lines of treatment, ie, both experimental and standard treatments. In detail, Case II includes 6 first-line cycles plus 4 cycles in the maintenance line, according to study NCT01907100, followed by standard treatment in the second and third line. Case III proposes standard treatments like in case I, plus 4 cycles of experimental treatment in the maintenance line according to NCT01358084. Case IV entails a mixed experimental strategy, with 6 first-line cycles plus 4 cycles in the maintenance line, according to study NCT01907100, another 4 cycles of experimental treatment in the second line according to study NCT01843374, followed by a final third line with standard treatment (note that 3 patients actually followed this mixed strategy). Case V proposes standard treatments in the first (6 cycles) and second line (4 cycles), with an experimental third line of 4 cycles according to NCT02194231.

This comparative analysis can shed new light on the impact of experimental treatments and/or mixed strategies on public finance. The results of the empirical analysis and the subsequent simulation are illustrated in the next section.

## Results

From January 1, 2014 to December 31, 2015, 102 patients with epithelioid MPM had their first medical examination in our hospital; 45 died before the end of data collection (30/06/2016) and constitute our selected sample. Note that we consider first access to the hospital, which is not necessarily the first medical examination after the MPM diagnosis, ie, patients might have been previously treated in other hospitals and then they chose to access our hospital for alternative treatments and/or opinions. Since MPM is a rare tumor, the authors believe that the considered sample of patients is representative for an empirical investigation of this specific case study.^1^ Table 2 summarizes some descriptive demographic statistics about the selected sample of patients, highlighting differences between males and females.

**Table 2:** Descriptive Demographic Statistics of the Selected Sample According to Patients’ Gender

**Variables**	**Female**	**Male**	**Total Sample**
Number of patients	17	28	45
% Of patients who moved to be treated (>50 km)	23.53%	21.43%	22.22%
Average distance between domicile and diagnostic center^a^	22.781	21.842	22.197
Average distance between domicile and treatment center^a^	44.641	34.927	38.597
% Of non-naïve patients	35.29%	42.86%	40.00%
% Of Caucasian patients (race)	94.12%	100.00%	97.78%
% Of Latino patients (race)	5.88%	0.00%	2.22%
Average age (at MPM diagnosis)^b^	64.059	68.821	67.022
Average survival (after MPM diagnosis)^c^	12.529	12.929	12.778

^a^ Distance expressed in kilometers. ^b^ Time expressed in years. ^c^ Time expressed in months.

In terms of how far the patients decide to move in order to be treated, Table 2 confirms the aforementioned hypothesis of greater expected effectiveness of the chosen treatments. On average, 22% of the selected sample traveled more than 50 km away from their domicile to receive an innovative treatment. In detail, 18 patients were treated by our oncology unit from the beginning (ie, their first medical access, after the MPM diagnosis) up to their death (ie, 40% of the sample). Taking the remaining 60% of the sample, the patients were treated in other medical centers before they first came to our hospital (ie, they had already received some chemotherapy cycles) and/or they chose another hospital where to be treated before their death.

Table 3 summarizes the average number of treatments offered to patients with MPM according to their gender, as well as relative average costs. Taking the total sample into account, the average cost of treating a patient with MPM is equal to € 8484.69. However, this is an average value which considers both the patients that had already been treated in other medical centers (ie, *non-naïve patient*) and the patients that left our hospital to be treated in other medical centers (ie,* exit patient*).

**Table 3:** Descriptive Economic Statistics of the Selected Sample According to Patients’ Gender

**Variables**	**Female**	**Male**	**Total Sample**
Average number of hospitalizations	0.529	0.357	0.422
Average number of chemotherapy cycles	4.324	5.137	4.830
Average number of follow-up visits	10.647	13.964	12.711
Average number of blood tests	9.941	13.214	11.978
Average number of instrumental exams	8.000	6.929	7.333
% Of patients involved in a first line trial	17.65%	10.71%	13.33%
% Of patients involved in a maintenance line trial	5.88%	7.14%	6.67%
% Of patients involved in a second line trial	11.76%	25.00%	20.00%
% Of patients involved in a third line trial	5.88%	3.57%	4.44%
Average total cost	7810.14	8894.24	8484.69
Average hospitalization cost	6301.10	3843.78	4826.71
Average chemotherapy cost	2993.03	4843.15	4144.22
Average monitoring cost	2241.02	2599.81	2464.27

Considering all the patients treated exclusively by the selected independent public hospital from the MPM diagnosis up to their death, the average total cost increases to € 15 866.44, with average chemotherapy cost equal to € 8813.40 and average monitoring cost equal to 4635.04. Note that the monitoring cost is given by the sum of follow-up medical visits, blood tests, and instrumental exams.

Supplementary file 1 shows additional economic descriptive statistics. In details, Table S1 highlights the per-cycle costs of the proposed antitumoral treatments; while Table S2 proposes other economic descriptive statistics related to the patients and their mobility process.

Table 4 sets out some descriptive statistics concerning the proposed dependent and independent variables of the empirical model. Note that a logarithmic transformation is applied to the *total cost* variable.

**Table 4:** Descriptive Statistics of Variables Adopted in the Empirical Analysis

**Type**	**Variable**	**Obs**	**Mean**	**SD**	**Min**	**Max**
Dependent variable	Total cost^a^	45	7.3047	2.7141	3.0301	10.7844
Control variables	Time (age)	45	67.0222	11.5552	28.0000	85.0000
	Time (survival)	45	8.7111	5.8954	0.0000	23.0000
	Non-naïve patient	45	0.4000	0.4954	0.0000	1.0000
	Exit patient	45	0.4222	0.4995	0.0000	1.0000
	Cancer stage	45	3.0444	0.6013	2.0000	4.0000
	Side effects tolerability	45	0.2667	0.4472	0.0000	1.0000
Explanatory variables	Hospitalizations	45	0.4222	0.6905	0.0000	2.0000
	First line (cycles)	45	0.3260	0.4221	0.0000	1.0000
	Maintenance (cycles)	45	0.7851	2.0997	0.0000	8.0000
	Second line (cycles)	45	1.2222	1.9672	0.0000	9.0000
	Third line (cycles)	45	0.5444	1.5368	0.0000	7.5000
	Standard treatments (quota)	45	0.4340	0.4557	0.0000	1.0000
	Maintenance study	45	0.0667	0.2523	0.0000	1.0000
	First-line study	45	0.1333	0.3438	0.0000	1.0000
	Second-line study	45	0.2000	0.4045	0.0000	1.0000
	Third-line study	45	0.0444	0.2084	0.0000	1.0000

^a^ Logarithmic transformation. Data were extracted from the dataset of the analyzed general hospital, applying the current reimbursement system of the regional system.

Table 5 shows the results of the adopted model (column 1), with the estimated coefficients and (in parentheses) the relative robust standard errors. Based on the suggested sensitivity analysis, additional model specifications are proposed (columns 2-5).

**Table 5:** OLS Regression Model - Robust Option: Cost Components of Treating MPM

**Variables**	**(1)**	**(2)**	**(3)**	**(4)**	**(5)**
	**Total Cost**^a^	**Total Cost**^a^	**Total Cost**^a^	**Total Cost**^a^	**Total Cost**^a^
Time (age)	-0.0354**				
	(0.0155)				
Time (survival)	0.118***				
	(0.0262)				
Non-naïve patient	0.557*	0.290			
	(0.284)	(0.392)			
Exit patient	-1.846***	-0.783**			
	(0.345)	(0.300)			
Cancer stage	-1.030***	-0.715***	-0.627***		
	(0.215)	(0.219)	(0.219)		
Side effects tolerability	-0.841**	-0.790**	-0.715**		
	(0.348)	(0.328)	(0.295)		
Hospitalizations	1.139***	0.940***	0.843***	0.570*	
	(0.189)	(0.214)	(0.308)	(0.320)	
First line (cycles)	1.283**	1.562**	1.378***	1.767***	
	(0.499)	(0.643)	(0.416)	(0.575)	
Maintenance (cycles)	-0.336***	-0.156	-0.176	-0.135	
	(0.0904)	(0.120)	(0.132)	(0.154)	
Second line (cycles)	-0.117	-0.00917	-0.00203	0.0407	
	(0.0785)	(0.0942)	(0.106)	(0.101)	
Third line (cycles)	-0.563***	-0.650***	-0.667***	-0.630***	
	(0.124)	(0.141)	(0.152)	(0.176)	
Standard treatments (quota)	3.131***	3.749***	4.167***	3.686***	4.313***
	(0.446)	(0.402)	(0.296)	(0.531)	(0.419)
First-line study	2.775***	2.800**	3.398***	2.826**	3.340***
	(0.735)	(1.120)	(1.207)	(1.167)	(0.489)
Maintenance study	2.212***	3.056***	3.429***	2.444***	1.871**
	(0.729)	(0.591)	(0.608)	(0.723)	(0.750)
Second-line study	2.998***	3.683***	4.067***	3.942***	3.108***
	(0.516)	(0.519)	(0.530)	(0.639)	(0.642)
Third-line study	2.084***	2.862***	3.361***	3.702***	4.119***
	(0.551)	(0.571)	(0.759)	(0.584)	(0.398)
Constant	9.846***	6.403***	5.647***	3.821***	4.156***
	(1.673)	(0.872)	(0.748)	(0.258)	(0.289)
Observations	45	45	45	45	45
R-squared	0.971	0.950	0.937	0.919	0.818

Abbreviations: MPM, malignant pleural mesothelioma; OLS, ordinary least squares. Robust standard errors in parentheses. *** *P* < .01, ** *P* < .05, * *P* < .1. ^a^ Logarithmic transformation.

By observing the results, we can see that the signs of the estimated coefficients do not change except in one single case (ie, the number of cycles in the second line). This coefficient, which is not statistically significant, is positive in the fourth model, while it is negative in the previous ones. Although there might be concerns regarding this variable, the sensitivity analysis confirms the robustness of our results.

Looking at the model proposed in column 1, the F-test confirms that it is statistically significant (*P* < .0000) and the R2 value is extremely high (ie, approximately 97% of the total cost variability is accounted for by the variables in the model). The distribution of residuals is also tested with good results, as is the correlation among covariates (ie, mean VIF displays a value equal to 4.01) and the *pairwise* correlation (ie, absence of major *collinearity* issues).

Considering the coefficients, all the dependent variables are statistically significant (*P* value at least lower than 0.1). The only concern is the number of cycles within the second-line treatment, although the *P* value obtained is not so poor (*P* < .15).

Referring to the simulated patient (section 2), Table 6 shows the results obtained by comparing the different clinical pathways adopted in the simulation.

**Table 6:** Expected Cost of Caring for Patients Affected by MPM According to Different Clinical Pathways

	**A**	**B**	**C**	**D**	**E**	**(A+B+C+D-E)**
**Clinical Pathways**	**Hospitalizations**	**First Line**	**Second Line**	**Third Line**	**Expected Grant**	**Standard Cost**
Case I	5353.55	8112.46	4030.73	718.24	-	18 214.99
Case II	2457.99	3320.73	1681.17	160.30	7300.00	320.18
Case III	10 388.63	19 823.95	7105.41	677.48	5500.00	32 495.47
Case IV	19 538.61	26 396.52	28 964.85	1274.19	36 050.00	40 124.18
Case V	15 685.68	23 769.15	11 809.87	8575.24	-	59 839.94

Abbreviation: MPM, malignant pleural mesothelioma.

According to our results, only one case affords significant savings in terms of public resources (ie, Case II with expected standard cost equal to € 320.18). Therefore, only the clinical pathway which includes study NCT01907100, followed by standard treatments in the second and third line (ie, Case II), has an expected *standard spending need* lower than a clinical pathway based exclusively on standard treatments (ie, Case I). In the other cases, the costs borne by the healthcare system to treat patients with experimental strategies grow exponentially. For example, in case III, even though the drugs are supplied free of charge and there is a grant for the required activities (€ 5500.00), the *standard spending need* in order to follow this strategy is higher than the expected cost of adopting the common treatments (€ 32 495.47 vs. € 18 214.99). How can these results be interpreted?

## Discussion

As indicated by our results, there is a clear saving of public resources in the case of first-line treatments, while the following lines are characterized by a higher economic burden. This is mainly due to the current price of chemotherapy drugs, operative costs and toxicity of experimental treatments. Indeed, the cost of first-line chemotherapy is considerable, whereas the cost of the subsequent lines is lower. At the same time, there was a higher toxicity level at second and at third line, as well as a higher number of diagnostic and blood examinations. The economic gap between traditional and experimental treatments is crucial in the compensation of the higher operating costs due to the increased number of medical activities required by a clinical trial, as well as the necessary treatments to face the unexpected toxicity. For example, considering a patient with body surface area (BSA) equal to 1.8 mt^2^, a cycle of cisplatin plus pemetrexed costs € 2398.79 (first-line treatment), while a cycle of Carboplatin plus pemetrexed costs € 2408.10 (first-line treatment). In the second or third line, the same patient might be treated with vinorelbine, costing € 71.50 for every cycle, or gemcitabine, costing € 37.00 for every cycle, or carboplatin plus gemcitabine, costing € 59.27 for every cycle (see Table S2 in Supplementary file 1). At the same time, the number of medical investigations for a patient involved in an experimental second-line treatment would increase dramatically. For example, assuming 6 cycles of chemotherapy and a patient involved in a second-line trial (ie, NCT01843374), we can expect the patient to access the hospital 12 times, with blood tests and medical checks, as well as 2 computerized axial tomography (CAT) scans, 1 positron emission tomography (PET) and 7 X-rays. Considering the same patient but treated according to a traditional clinical second-line protocol (ie, vinorelbine), we can expect the patient to access the hospital 12 times, with blood tests and medical checks, but the number of other diagnostic investigations would be lower (ie, 1 CAT scan, 3 X-rays and 1 electrocardiogram). If we consider the toxicity level, at present we cannot support the hypothesis that experimental drugs at second and third level have lower tolerability, since we need to wait for the publication of official reports by the sponsors about collected toxicity data. However, we cannot reject this hypothesis *a priori* since it is coherent with the current knowledge.

Therefore, our results clearly show the economic impact of experimental treatments in subsequent lines, ie, after the first (expensive) line. Indeed, these experimental treatments might lead to an increase in operating and hospitalization costs, without a significant drug cost saving. Moreover, the higher operating costs are not completely compensated for by the grants offered, which means that hospital managers should carefully consider whether to be involved in a clinical trial, comparing grants with all expected costs. What about the other variables?

According to our results, we can expect a patient’s health status upon first medical access to be a significant driver of cost, since an expected higher number of medical treatments will be provided by the regional healthcare system. Indeed, the older the patient and/or the more advanced the stage of cancer, the lower the final expected costs borne by the regional healthcare system (ie, statistically significant negative coefficients), which is mainly due to the lower number of medical activities in terms of diagnostic examinations. This result is coherent with the survival time (ie, statistically significant positive coefficient), that is to say, the longer the expected patient’s survival time, the higher the expected costs borne by the regional healthcare system. Therefore, although the current scientific knowledge does not provide a strategy to defeat MPM, patients are involved in very intensive clinical protocols, which become extremely expensive. Moreover, mainly due to the high toxicity level of the drugs used, hospitalization is needed multiple times, which can be considered one of the main drivers of cost in oncology units (ie, statistically significant positive coefficient). This hypothesis is supported by the variable adopted to capture side effects tolerability, which can reduce the final cost of treating patients with MPM (ie, statistically significant negative coefficient). Indeed, the ability to tolerate the high toxicity of chemotherapy drugs can be crucial in limiting the need for supportive care.

## Study Limitations

The main limits of this study concern the sample, which is quite small (ie, 45 patients), and the available information. On the one hand, the sample comprises the most critical patients, that is to say, the patients who died before the end of data collection. On the other hand, many patients were involved in clinical trials and complete information is not currently available (eg, whether these subjects received placebo or experimental treatments). Another limit relates to the proposed hypothesis behind the adopted costs. Indeed, the fees used as reimbursements by the Local Health Authorities represent our reference costs, assuming that a competitive market drives the health providers toward those standard costs (ie, those efficiency levels). Obviously, there are risks of overestimation in our results if the real cost is lower than the fee (ie, the hospital is more efficient than the standard level) or underestimation if the real cost is higher than the fee (ie, the hospital is less efficient than the standard level). Therefore, these results should be taken with a degree of caution and they cannot be generalized.

Despite the above limitations, this study may represent a solid first step for a broader and deeper analysis of the topic. Indeed, our work can stimulate the debate on the economic impact of human experimentation among policy-makers, hospital managers, and physicians.

Future research will be aimed at confirming the results of this pilot study by involving other hospitals specialized in MPM care, thus significantly increasing the sample size. At the same time, the authors will try to extend the methodology applied here to include other types of cancer (eg, head and neck cancer), so as to validate the proposed approach and highlight whether differences might exist.

## Conclusion

This is an empirical, retrospective analysis focusing on a reference hospital specialized in MPM care. This hospital is a forefront medical reference center in the North West of Italy, assisting MPM patients from the main polluted area (ie, Casale Monferrato), as well as other relevant areas (ie, Cavagnola and Broni). In detail, we conducted a retrospective cost analysis on all the patients with MPM who first accessed the hospital between 2014 and 2015; collecting data about their diagnostic pathways and active treatments, as well as the related official fees for each procedure. Then, using a multiple regression model, we estimated the overall expected cost of a patient with MPM treated in our hospital, which would be borne by the regional healthcare system based on the clinical pathways.

Our results confirm that drug cost avoidance might be positive but, in order to properly estimate the economic impact of clinical trials on public healthcare systems, we also need to consider alternative standard treatments along the entire clinical pathway of the research subjects. Indeed, our results indicate that experimental trials might be a solution to reduce the economic burden placed on public healthcare systems only in the case of first-line treatments, where the cost of chemotherapy is substantial. For what concerns second-line and third-line treatments, even if the drugs were supplied to the medical centers by the sponsor for free, there would be no opportunities to save money since, as our analysis shows, the regional healthcare system would have to bear higher costs. This means that, in the second and third line, although experimental treatments might represent an opportunity to increase scientific knowledge and the research subjects’ expected survival time, society has to share with pharmaceutical companies a portion of the costs needed to test the proposed innovations and, by doing so, to collect clinical evidence. Therefore, in order to expand medical knowledge, society needs not only to accept the pharmaceutical companies’ market power (ie, monopoly) and a part of the adverse risks linked to clinical research^9^ but also to bear the additional economic costs related to the testing phase. Moreover, patient costs should not be forgotten. According to current literature, clinical trials involving cancer patients entail the added cost of more frequent clinical checkups and additional tests.^22,32,33^ Only if the patients accept to bear all expected adverse risks and additional costs, signing the informed consent form, and the hospital management decides to face the additional economic burden, signing the contract, will the investment be made, since a profit is foreseeable. On the other hand, pharmaceutical companies will deal with all unexpected adverse events and bear the other portion of the costs needed to treat the research subjects (ie, supplying free drugs and grants). The risk and cost sharing idea relies on the distribution of adverse events and costs among the parties (ie, patients, hospitals, and pharmaceutical companies), and the present study sheds some new light on this relevant topic. Society should consider its involvement in clinical trials with particular attention, properly balancing costs and risks, but this is the price to be paid if we want to win the battle against MPM.

## Ethical issues

This is a retrospective cost analysis on anonymous data, previously collected by the hospital, without formal ethical approval according to national law. Patients’ informed consents have been collected by the hospital before treatments and clinical procedures.

## Competing interests

Authors declare that they have no competing interests.

## Authors’ contributions

The leading authors are IR and NG, with the key support of FG in data analysis. RL supported the research team in data collection, while the other two authors have made equal contributions in reviewing the manuscript.

## Authors’ affiliations

^1^Scientific Promotion, General Hospital of Alessandria, Alessandria, Italy. ^2^Department of Management, University of Turin, Turin, Italy. ^3^Research Institute on Sustainable Economic Growth, National Research Council of Italy, Moncalieri, Italy. ^4^Oncology Unit, General Hospital of Alessandria, Alessandria, Italy.

## Supplementary files

Supplementary file 1 contains Tables S1-S2.

## Key messages

**Implications for policy makers**

- Drug cost avoidance might be positive but, in order to properly estimate the economic impact of clinical trials on public healthcare systems, we also need to consider alternative standard treatments along the entire clinical pathway of the research subjects.
- Even if experimental treatments might represent an opportunity to increase scientific knowledge and the research subjects’ expected survival time, society has to share with pharmaceutical companies a portion of the costs needed to test the proposed innovations and, by doing so, to collect clinical evidence.

**Implications for the public**

The current economic constraints cause hospital management to use the available public resources as rationally as possible. At the same time, there is the necessity to improve current scientific knowledge. Pharmaceutical clinical research might be an opportunity to face these issues, especially in those rare tumors characterized by dismal prognosis and significant cost of chemotherapy drugs.

According to the results of this work, experimental trials might be a solution to decrease the economic burden for the public healthcare system only in some cases. Therefore, in order to expand medical knowledge, society needs not only to accept the pharmaceutical companies’ market power (ie, monopoly) and a part of the adverse risks linked to clinical research but also to bear the additional economic costs related to the testing phase. This is the price to be paid if we want to win the battle against rare tumors.
