# Supplementary material for: The Economic Impact of Clinical Research in an Italian Public Hospital: The Malignant Pleural Mesothelioma Case Study
Source: Int J Health Policy Manag. 2018 Feb 18;7(8):728–37. doi: 10.15171/ijhpm.2018.13 (PMC6077275; doi:10.15171/ijhpm.2018.13)
Supplement: Supplementary file 1 — contains Tables S1-S2. [file ijhpm-7-728-s001.pdf]

Table S1. Costs of Drugs According to Potential Clinical Pathways for Patients With MPM

Line of Chemotherapy	Drugs	Type of Treatment	Chemotherapy Cost Per-Cycle[a]
First line	Cisplatin and Pemetrexed	Standard	€ 2398.79
	Carboplatin and Pemetrexed	Standard	€ 2408.10
	Cisplatin and Pemetrexed and Nintedanib (or placebo)	Experimental	€ 0.00
Maintenance line	Nintedanib (or placebo)	Experimental	€ 0.00
	NGR-hTNF (or placebo)	Experimental	€ 0.00
Second line	Gemcitabine	Standard	€ 37.00
	Vinorelbine	Standard	€ 71.50
	Carboplatin and Gemcitabine	Standard	€ 59.27
	Trabectedina	Experimental	€ 0.00
Third line	Gemcitabine	Standard	€ 37.00
	Vinorelbine	Standard	€ 71.50
	Tremelimumab	Experimental	€ 0.00

Abbreviation: MPM, malignant pleural mesothelioma.

[a] Considering a patient with Body Surface Area (BSA) equal to 1.8 mt^2

Table S2. Descriptive Economic Statistics of the Selected Sample According to Patients' Gender

Variables	Patients Treated Completely[a]	Patients Treated Partially[b]	Total Sample
Number of patients	18	27	45
Average total cost	15 866.44	3563.52	8484.69
Average hospitalization cost	4976.00	1391.43	4826.71
Average chemotherapy cost	8813.40	1031.43	4144.22
Average monitoring cost	4635.04	1017.09	2464.27

[a] Patients treated from their MPM diagnosis to their death.

[b] Patients treated in our hospital and other medical centers.
